# Genomic Insights into *Vaccinium* spp. Endophytes *B. halotolerans* and *B. velezensis* and Their Antimicrobial Potential

**DOI:** 10.3390/ijms26146677

**Published:** 2025-07-11

**Authors:** Ingrida Mažeikienė, Birutė Frercks, Monika Kurgonaitė, Neringa Rasiukevičiūtė, Irena Mačionienė

**Affiliations:** 1 Department of Orchard Plant Genetics and Biotechnology, Institute of Horticulture, Lithuanian Research Centre for Agriculture and Forestry, Kaunas District, LT-54333 Babtai, Lithuania; birute.frercks@lammc.lt (B.F.); monika.kurgonaite@lammc.lt (M.K.); 2 Research Laboratory of Microbiology, Food Institute, Kaunas University of Technology, Radvilėnų Avenue 19, LT-50254 Kaunas, Lithuania; irena.macioniene@ktu.lt; 3 Laboratory of Plant Protection, Institute of Horticulture, Lithuanian Research Centre for Agriculture and Forestry, Kaunas District, LT-54333 Babtai, Lithuania; neringa.rasiukeviciute@lammc.lt

**Keywords:** bacteria, fungi, nutritional preferences, resistance, horizontal gene transfer

## Abstract

Plant microbiota contributes to nutrient absorption, and the production of hormones and vitamins, and plays a crucial role in responding to environmental stress. We hypothesized that *Vaccinium* spp. harbour a unique microbiota that enables them to coexist in extreme environments such as saline, nutrient-poor, and waterlogged conditions. Upon examining *Bacillus* spp. endophytes isolated from blueberries, cranberries and lingonberries in vitro, we identified *B. halotolerans* (Bil-LT1_1, Bil-LT1_2) and *B. velezensis* (Cran-LT1_8, Ling-NOR4_15) strains that inhibit the growth of five pathogenic fungi and five foodborne bacteria. Whole-genome sequencing provided insights into genome organization and plasticity, helping identify mobile elements and genes potentially acquired through horizontal gene transfer. Functional annotation identified genes associated with plant colonization, stress tolerance, biocontrol activity, and plant growth promotion. Comparative genomic analyses revealed key biosynthetic gene clusters (BGCs) responsible for producing antifungal metabolites, including lipopeptides and polyketides. Genes supporting plant nutrition, growth, and environmental adaptation were present also in these strains. Notably, isolated endophytes exhibited particularly high levels of genomic plasticity, likely due to horizontal gene transfer involving gene ontology (GO) pathways related to survival in polymicrobial and foreign environments.

## 1. Introduction

Endophytes are microorganisms that colonize internal plant tissues without causing immediate harm to the host. However, not all bacterial endophytes are beneficial—some may behave as latent pathogens, opportunists, or have neutral effects, depending on environmental conditions, host immunity, or genetic factors [1]. According to in vitro studies, some endophytes have emerged as promising agents for biological control and plant growth promotion [2,3,4,5]. Conventional fungicide uses faces increasing challenges due to environmental concerns, the development of fungicide resistance, and rising consumer demand for organic products [6]. As a result, the exploration of plant-associated microbial communities for alternative strategies for disease management has gained momentum [7].

In recent years, the biocontrol potential of *Bacillus* spp. against phytopathogens has been extensively studied in an agroecological context [7,8,9,10,11]. Among bacterial endophytes, *Bacillus* species are particularly notable for their production of a wide range of bioactive secondary metabolites with antimicrobial properties. Metabolites, including lipopeptides and polyketides, are typically encoded within biosynthetic gene clusters (BGCs) that can be identified through genome mining. Individual strains of *B. subtilis*, *B. amyloliquefaciens*, and *B. velezensis* have been widely studied for their production of antimicrobial compounds such as iturins, surfactins, and fengycins [8,9,10,11]. These lipopeptides disrupt fungal membranes and inhibit spore germination and hyphal growth of fungi. Unlike chemical fungicides, a key advantage of bacterial biocontrol agents is their ability to simultaneously promote plant growth and development.

The genus *Vaccinium* spp., which includes wild berries crop such as blueberries, bilberries, and cranberries, is adapted to a wide range of growing conditions in northern regions [12]. *Bacillus* endophytes from blueberry have previously been investigated for their antagonistic activity against *Monilinia vaccinii-corymbosi* [13], genetic-level studies remain scarce. Comparative genomic approaches have proven effective for identifying functionally important genes in other *Bacillus* species [14], but remains underexplored in endophytes from ericaceous hosts such as *Vaccinium* spp. Recent advances in whole-genome sequencing (WGS) and bioinformatics now allow for high-resolution insights into microbial genetic potential [15,16]. Despite the recognized benefits of endophytes, relatively few studies have investigated their diversity and functional capabilities in *Vaccinium* spp. [17,18]. Moreover, genomic studies of endophytic *Bacillus* strains from various hosts remain highly relevant, particularly in relation to bacterial genetic diversity shaped by horizontal gene transfer (HGT) within tightly associated microbial communities [19,20,21]. Identifying and characterizing such strains is critical for the development of effective and safe biocontrol agents specialized for plants and crop conservation.

This study aims to comprehensively characterize the genomes of *B. halotolerans* and *B. velezensis* endophytes isolated from *Vaccinium* spp., with particular focus on their genetic potential and the mechanisms underlying their antimicrobial activity.

## 2. Results

### 2.1. Antagonistic Activity and Nutritional Preferences Test In Vitro

The primary objective of the in vitro screening was to identify endophytic strains with antagonistic activity against bacterial and fungal pathogens, as candidates for subsequent genomic and functional characterization.

Four *Bacillus* isolates (Bil-LT1_1, Bil-LT1_2, Cran-LT1_8 and Ling-NOR4_15) demonstrated antagonistic activity in vitro against tested phytopathogenic fungi and foodborne pathogenic bacteria (Table 1). Fungal growth inhibition was observed in vitro on potato dextrose agar (PDA) plates for isolates from genera *Botrytis*, *Fusarium* and *Monilinia* (Figure S1). All bacterial strains had a negative effect on fungal growth, with suppression observed in all cases, though the fungi occupied different areas of the Petri plates Cran-LT1_8 exhibited the strongest antifungal activity, inhibiting the growth of *B. cinerea*, *M. fruticola*, and *M. laxa* by the highest percentages. *M. fructigena* did not grow at all under the influence of Bil-LT1_2. Ling-NOR4_15 most effectively inhibited the growth of *F. culmorum*.

Similarity, antibacterial activity was assessed on Luria-Bertani (LB) agar plates, inhibition zones were observed against five foodborne pathogens. Among the tested isolates, strain Cran-LT1-8 exhibited the strongest antibacterial activity. *S. aureus* subsp. *aureus* ATCC 25923 and *L. monocytogenes* ATCC 13932 were the most sensitive to the antibacterial effects of the endophytes. Strain Ling-NOR4_15 showed no inhibitory effect on *E. coli* ATCC 25922. Strains Bil-LT1_1 and Bil-LT1_2 did not inhibit the growth of *E. coli* ATCC 25922 or *S. enterica* subsp. *enterica serovar Typhimurium* ATCC 13932.

**Figure 1:**
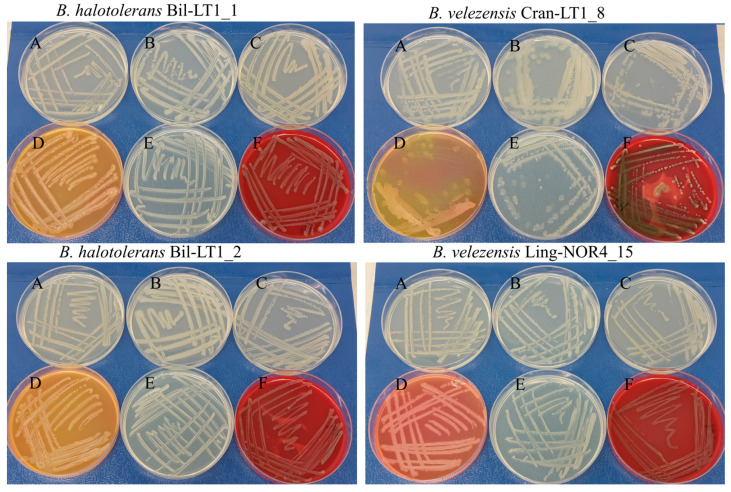
*Bacillus* spp. strains on different nutrition medium. A—Luria-Bertani agar (LB); B—Plate Count Agar (PCA); C—Tryptic Soy Agar (TSA); D—Mannitol egg Yolk Polymyxin agar (MYP); E—Nutrient agar (NA); F—Sheep Blood agar (SBA).

The growth behaviour of *Bacillus* spp. strains was assessed on six different nutrient media to assess their metabolic versatility and morphological characteristics (Table 1 and Figure 1). All tested strains demonstrated good growth on LB agar, indicating non-fastidious metabolism and the ability to utilize a wide range of nutrients (Figure 1, A). On Plate Count Agar Medium (PCA), a low-nutrient medium commonly used for total viable counts (Figure 1, B), all strains grew, with Bil-LT1_2 forming denser colonies and Cran-LT1_8 producing larger, slightly mucoid colonies. These observations suggest environmental resilience and adaptability under nutrient-limited conditions. On Tryptic Soy Agar (TSA), a rich medium supports a broad spectrum of microbial growth, Bil-LT1_1 displayed yellow pigment production, while Cran-LT1_8 showed reduced colony density (Figure 1, C).

On Mannitol Egg Yolk Polymyxin (MYP) agar, used to differentiate members of the *B. cereus* group (Figure 1, D), Bil-LT1_1 and Bil-LT1_2 showed an orange/yellow color shift, suggesting mannitol fermentation. Cran-LT1_8 displayed partial yellow coloration and inhibited growth, suggesting weak lecithinase activity and possible monnitol fermentation. In contrast, Ling-NOR4_15 exhibited a pinkish hue, indicating no mannitol fermentation. Growth on Nutrient Agar (NA), a general-purpose medium (Figure 1, E), was uniform for strains Bil-LT1_1, Bil-LT1_2 and Ling-NOR4_15. Cran-LT1_8 showed reduced growth and occasionally slightly mucoid colonies on NA plates. Hemolytic activity was assessed using Sheep Blood Agar (SBA) (Figure 1, F). Strains Bil-LT1_1, Bil-LT1_2 and Ling-NOR4_15 produced clear zones of hemolysis (beta-hemolysis) against a red background. Cran-LT1_8 exhibited both alpha- and beta-hemolytic activity, characterized by greenish partial hemolysis (alpha) and areas of complete clearing (beta).

### 2.2. Genetic Characteristics

The whole genome sequencing of the endophytic *Bacillus* spp. isolates (BioProject PRJNA991431, NCBI) resulted in high-quality draft genomes. The main statistics of sequencing, read mapping, and assembly are presented in Table 2. The genome sizes are as follows: Bil-LT1_1—4,259,074 bp, Bil-LT1_2—4,261,465 bp, Cran-LT1_8—3,958,789 bp and Ling-NOR4_15—3,987,686 bp. The corresponding GC contents were 45.54%, 46.19%, 48.48% and 48,71%, respectively.

ANI analysis showed 99.50% and 99.82% identity with *B. halotolerans* for Bil-LT1_1 and Bil-LT1_2, and 98.01% and 97.95% with *B. velezensis* for Cran-LT1_8 and Ling-NOR4_15 (Table 2, Figure S2), confirming species-level identity. Minor sequence differences likely reflect strain-specific traits, including variations in resistance genes, metabolism, or mobile elements.

Both *B. halotolerans* strains shared an identical set of 13 antibiotic resistance genes (Table S1). In *B. velezensis*, seven antibiotic resistance genes were detected per strain. Notably, the tetracycline resistance gene *tet*(45) was found exclusively in strain Cran-LT1_8.

A similar number of virulence factors (VFs) were identified across all strains, though species-specific differences were noted: 39 in *B. halotolerans* and 38 in *B. velezensis* (Table S2). Fourteen VFs were common to all strains. VF classes associated with invasion and motility, such Flagella (Burkholderia) and Flagella (Campylobacter), were found exclusively in *B. halotolerans*. In contrast, *B. velezensis* strains carried VFs belonging to the Cell Surface Components class, including the GPL locus and Trehalose-recycling ABC transporter (Mycobacterium type).

Predicted pathogenicity scores were low, ranging from 0.0511 to 0.0942. All genomes were classified as non-pathogenic to humans.

Using antiSMASH, biosynthetic gene clusters (BGCs) responsible for secondary metabolites production were identified (Table S3). *B. velezensis* exhibit higher biosynthetic potential, with 20 BGCs in Cran-LT1_8 and 21 in Ling-NOR4_15. Although *B. halotolerans* strains (Bil-LT1_1 and Bil-LT1_2) each contain 14 BGCs, they are particularly valued for traits enhancing plant stress tolerance (e.g., to salt and drought), further supporting their biocontrol potential (Table 3).

All strains produced known antifungal lipopeptides, including plipastatin, bacillomycin D, and fengycin, and antibacterial compounds such as bacillaene, surfactin, and bacilysin, together contributing to their multi-targeted antagonistic activity. Unique metabolites were also identified. *B. halotolerans* strains contained thailanstatin A and subtilosin A, that likely enhances their fungicidal and bactericidal properties. *B. velezensis* strains featured BGCs for butirosin A/butirocin, molybdenum cofactor, difficidin, and macrolactin H, expending their efficacy against both Gram-positive and Gram-negative pathogens, in our case *S. enterica* subsp. *enterica serovar Typhimurium* ATCC13932. *B. velezensis* Ling-NOR 4_15 carried the BGCs for plntazolicin, a ribosomally synthesized and post-translationally modified peptide (RiPP). *B. velezensis* Cran-LT1_8 encoded the lantibiotic mersacidin and the lipopeptides rhizomides A, B and C, further boosting its antagonistic potential to E. coli ATCC 25922. A comprehensive overview of all identified BGCs, based on antiSMASH analysis, is provided in Table S3.

The number of protein-coding genes assigned to KEGG orthology (KO) categories is shown in Table S4. The percentage of annotated genes mapped to KEGG pathways ranges from 57.0% to 60.8%. Notable differences in pathway profiles were observed between the *B. velezensis* strains Cran-LT1_8 and Ling-NOR4_15, while the *B. halotolerans* strains Bil-LT1_1 and Bil-LT1_2 exhibited nearly identical profiles. Overall, KEGG pathway distributions were consistent across all strains, except in the terpenoids and polyketides biosynthesis category.

Both *B. halotolerans* strains contained the genes *srfAA*, *srfAB*, *srfAC*, *fenB*, *fenD*, while the gene *fenE*, involved in fengycin synthesis, was detected only in Bil-LT1_2. Differences in nonribosomal peptide synthetase (NRPS) biosynthesis was particularly evident between *B. velezensis* strains. Ling-NOR4_15 carried complete gene clusters for the synthesis of surfactin (*srfAA*, *srfAB*, *srfAC*), iturin (*ituA*, *ituB*, *ituC*) and fengycin (*fenA*, *fenB*, *fenC*, *fenD*, *fenE*). In contrast, strain Cran-LT1_8 possessed a complete iturin cluster but lacked key genes for surfactin (*srfAA*, *srfAB)* and fengycin (*fenC*, *fenE*).

All investigated *Bacillus* strains exhibited plant growth-promoting (PGR) genetic features, which may indirectly enhance plants resistance to pathogens by improving overall plant health and vigour. These PGR-associated genes summarized in Table 4, are involved in nutrient acquisition, stress tolerance, phytohormone modulation, signal molecule production, and direct promotion of plant growth.

**Figure 2:**
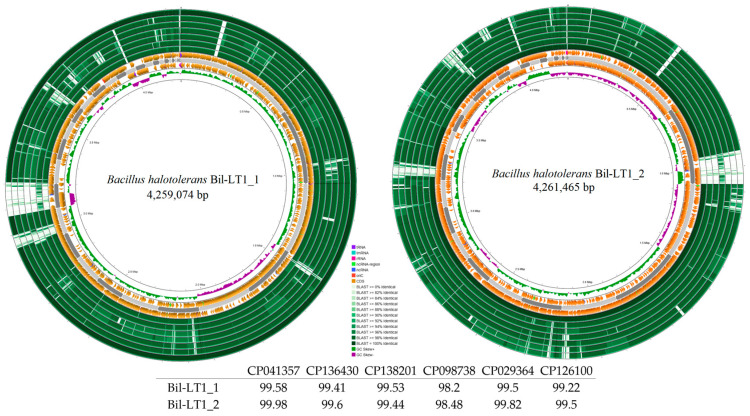
Circular genome maps *B. halotolerans* strains. Bakta annotation performed. Starting from the outermost ring: Ring 1: NZ_CP041357 Ring 2: NZ_CP136430 Ring 3: NZ_CP138201.1 Ring 4: NZ_CP098738.1 Ring 5: NZ_CP110264 Ring 6: NZ_CP126100 Ring 7: NZ_CP029364 8: Bakta Annotation (+) Backbone (Contigs) Ring 10: Bakta Annotation (−) Ring 11: GC Skew.

**Figure 3:**
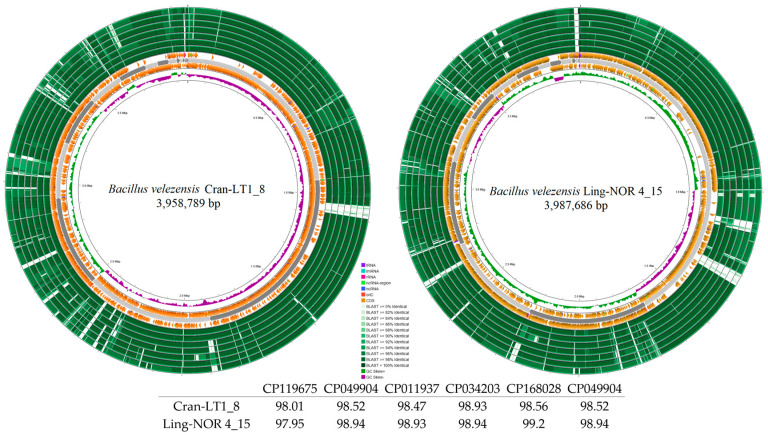
Circular genome maps *B. velezensis* strains. Bakta annotation of *Bacillus* spp. performed. Starting from the outermost ring: Ring 1: NZ_CP119675.1 Ring 2: CP049904 Ring 3: NZ_CP011937.1 Ring 4: CP034203 Ring 5: NZ_CP168028.1 Ring 6: NZ_CP026610.1 Ring 7: NZ_CP009679 Ring 8: Bakta Annotation (+) Backbone (Contigs) Ring 10: Bakta Annotation (−) Ring 11: GC Skew.

Genome mapping of the newly identified endophytic strains is shown in Figure 2 and Figure 3. Comparative analysis using Blast+ v2.16.0 against closely related strains in NCBI (with annotated antibacterial activity) revealed differences among strains. *B. halotolerans* strains Bil-LT1_1 and Bil-LT1_2 showed > 98% sequence identity with seven *B. halotolerans* reference genomes. The highest similarities were observed with strain F41-3 (CP041357), at 99.58% and 99.98%, respectively (Figure 2). *B. velezensis* strains Cran-LT1_8 and Ling-NOR4_15 aligned with seven *B. velezensis* reference genomes (Figure 3). *B. velezensis* Cran-LT1_8 exhibited sequence identity ranging from 98.01% to 99.23%, with the closest match to strain JS25R (CP009679). *B. velezensis* Ling-NOR4_15 showed identity value from 97.95% to 99.41%, with the highest similarity to strain CGMCC11640 (CP026610).

### 2.3. Horizontal Gene Transfer

Horizontal gene transfer (HGT) is a key evolutionary mechanism in bacteria, enabling the acquisition of new traits such as antibiotic resistance, metabolic capabilities, and virulence. Identifying and annotating horizontal gene transfer elements (HGTEs) is essential for understanding bacterial adaptation and genome plasticity. Using comparative genomics and annotation tools (AlienHunter), between 28 to 123 putative HGT regions were identified in the genomes of *B. halotolerans* and *B. velezensis* strains (Table 4).

**Figure 4:**
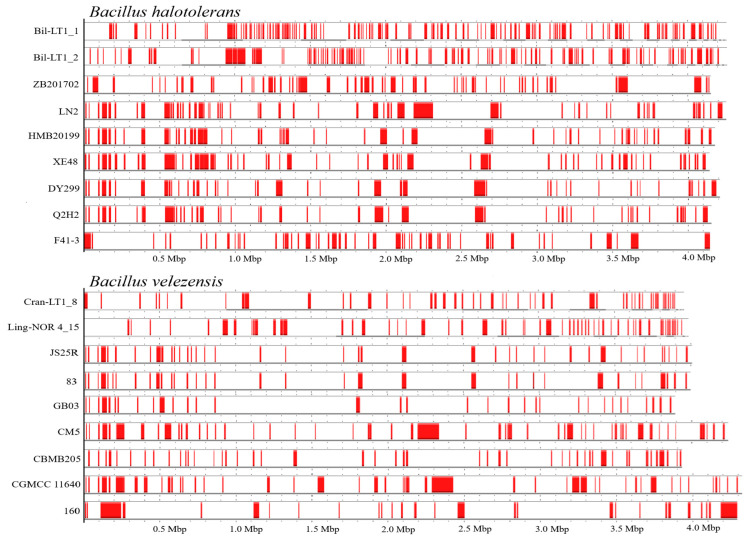
Alignment of Horizontal Gene Transfer Elements (HGTEs) profiles in *B. halotolerans* and *B. velezensis* strains (according to data from AliensHunter, HGTEs marked in red). The accession numbers of strains in NCBI are displayed in Table 4.

HGT regions size varied from 3808 to 142,965 bp. These regions often show atypical GC content and are flanked by mobile genetic elements (e.g., transposases, integrases, prophages), suggesting acquisition from external sources. Interestingly, even genetically similar bacterial strains showed hight variability in the number of HGTEs. Genome features annotation and HGTEs profiles are summarized in Table 5 and Figure 4.

HGTEs significantly contribute to genome flexibility. Newly identified *B. halotolerans* endophytes contained a notably high number of HGTEs-123 in Bil-LT1_1 and 107 in Bil-LT1_2, despite similar genome size and identity. For comparison, previously sequenced *B. halotolerans* strains carried from 46 to 61 HGTEs, with HMB20199 and Q2H2 strains (known for antagonistic activity) containing fewer HGTEs than 54 and 49, respectively. HGTEs size varied widely; the shortest (4055 bp) and longest (128,846 bp) were observed in *B. halotolerans* strain LN2.

*B. velezensis* strains showed greater genome size variation, but HGTEs ranged from 28 to 56 among strains (Table 5 and Figure 4). *B. velezensis* strains Cran-LT1_8 and Ling-NOR4_15 harbored 50 and 56 HGTEs, respectively, placing them among the top strains in genome plasticity. Notably, *B. velezensis* strain CM5—also isolated from a peat-rich environment—exhibited the same HGTEs count (56), reinforcing the idea that ecological niches like peat can shape bacteria genome evolution. Endophytes from long-established communities in wild *Vaccinium* spp. leaves, especially *B. halotolerans*, displayed elevated genetic plasticity. HGTEs were identified based on hallmark genes, conserved domains, and atypical sequence features.

The Venn diagram illustrate shared and unique genes in HGTEs among strains (Table 6 and Table S5). Despite 99.99% ANI identity and nearly identical in KEEG profiles, *B. halotolerans* strains Bil-LT1_1 and Bil-LT1_2 differed in HGTE content, containing 39 and 125 unique CDSs, respectively. Likewise, *B. velezensis* strains Cran-LT1_8 and Ling-NOR4_15 had 65 and 62 unique HGTE-associated genes. Antibiotic resistance genes, virulence factors, and NRPS biosynthetic gene clusters (e.g., bacillibactim, fengycin, plipastatin, mersacidin) were also transferred via HGTEs. These additions expand the strains’ antimicrobial capabilities and adaptability.

**Figure 5:**
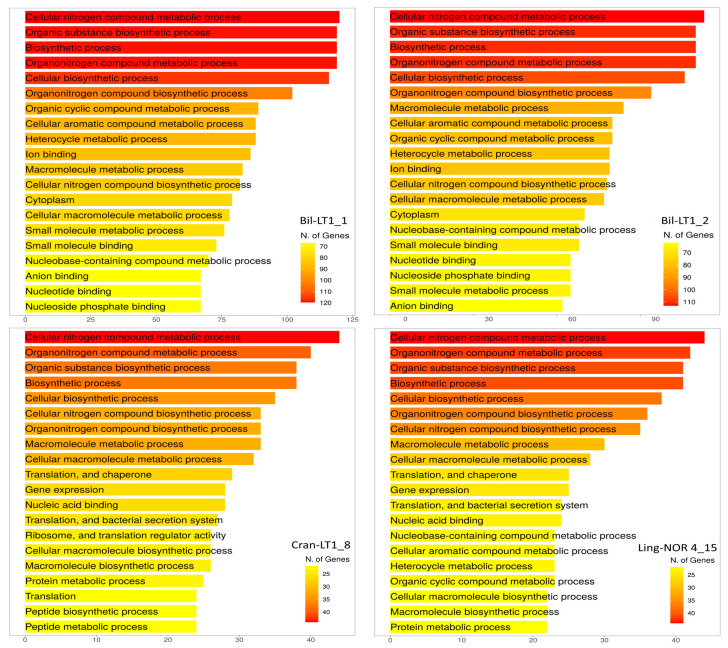
Number of genes in Gene Ontology (GO) pathways (ShinyGO 0.82).

Functionally, HGTEs enhance bacterial fitness by enabling antimicrobial production, nutrient acquisition, and resistance to environmental stressors. Gene Ontology (GO) analysis revealed that most HGTE-associated genes fell into five dominant pathways—Cellular Nitrogen Compound Metabolic Process (GO:0034641), Organic Substance Biosynthetic Process (GO:1901576), Biosynthetic Process (GO:0009058), Organonitrogen Compound Metabolic Process (GO:1901564) and Cellular Biosynthetic Process (GO:0044249) (Figure 5). Other notable pathways include Macromolecule Metabolic Process (GO:0043170) and Cellular Aromatic Compound Biosynthetic Process (GO:0019438). These functions support the adaptive potential of *B. halotolerans* strains Bil-LT1_1 and Bil-LT1_2, and *B. velezensis* strains Cran-LT1_8 and Ling-NOR4_15, enabling their survival and competitiveness in nutrient-poor, stress-prone environments such as those occupied by *Vaccinium* spp.

## 3. Discussion

Wild plant species, particularly those thriving in extreme or nutrient-poor environments, represent underexplored reservoirs of microbial diversity. These habitats foster unique endophytic communities that are precisely adapted to host physiology and ecological pressures [22]. In this context, the current study provides a detailed genetic characterization of four *Bacillus* spp. endophytes isolated from *Vaccinium* spp. genus including bilberry, lingonberry, and cranberry, all adapted to acidic, oligotrophic soils. The data emphasized antimicrobial potential, diversity of secondary metabolites synthesis clusters, plant growth-promoting (PGP) genes, and genomic plasticity.

Whole-genome sequencing and comparative analyses of *Bacillus* strains Bil-LT1_1, Bil-LT1_2, Cran-LT1_8, and Ling-NOR4_15 highlight their multifunction potential as both biocontrol agents and plant mutualists. Notably, the genome mapping and comparative BLAST+ v2.16.0 analysis of the newly isolated endophytic strains revealed high genomic similarity to *B. halotolerans* (Bil-LT1_1 and Bil-LT1_2) and *B. velezensis* (Cran-LT1_8, and Ling-NOR4_15) environmental isolates, and highlighting their taxonomic placement and suggesting potential bioactivity consistent with related strains.

The ability of *Bacillus* spp. isolates to thrive on diverse media under nutrient-limited conditions underscores their ecological plasticity, a hallmark of successful endophytes capable of adapting to the physicochemical constraints of plant tissues [23]. This adaptability is complemented by distinct phenotypic features including variations in pigment production, haemolytic activity, and colony morphology implying possible divergence in colonization strategies or niche specialization among the strains. While haemolytic activity, particularly beta-hemolysis, often raises concerns in clinical microbiology, in a plant context may indicate competitive or symbiotic enzymatic functions, involved in surfactin production, biofilm formation, or modulation of host immune responses [24]. The strains in this study exhibited hemolytic traits without predicted pathogenicity, reinforcing their potential safety and utility as biocontrol agents. Predicted pathogenicity results indicates these isolates are non-pathogenic to humans, and their application in the biological control enhancement of crops and food safety can be analysed in the perspective. Importantly, Bacillus strains are already being explored for applications in food safety and prebiotic formulations [25].

Plant endophytic *Bacillus* species are widely recognized for their antagonistic activity against phytopathogenic fungi, attributed to their abilities to produce diverse antimicrobial compounds such as lipopeptides and polyketides [2]. Four isolates presented in this study showed in vitro inhibition against five fungi isolate including genera *Botrytis*, *Fusarium*, *Monillinia*. These findings align with previous reports on the antifungal efficacy of *B. velezensis* and *B. halotolerans* against pathogens from genus *Botrytis* and *Fusarium* [21,26,27]. The negative effect of *B. velezensis* on *Moniliania* spp. pathogens growth in vitro has been established [3], however, the antagonistic effect of *B. halotolerans*, and its very intense antifungal effect, was unexpected.

Cran-LT1-8 showed the strongest antibacterial activity against five foodborne pathogens, particularly against *S. aureus* and *L. monocytogenes*, suggesting its potential as a biocontrol agent against Gram-positive pathogens. In contrast, no inhibition of *E. coli* or *S. enterica* was observed by any strain, likely due to the higher resistance of Gram-negative bacteria. The lack of activity by Ling-NOR4_15 and both *B. halotolerans* strains against these pathogens may reflect strain-specific differences in antimicrobial compound production.

Genomic mining with antiSMASH confirmed the presence of multiple secondary metabolite gene clusters for synthesis plipastatin, fengycin, and bacillomycin D, lipopeptides known to disrupt fungal membranes and inhibit mycelium growth. Moreover, additional bioactive compounds butirosin, mersacidin, plantazolicin, and rhizomides, enhance the antimicrobial arsenal of *B. velezensis* strains Cran-LT1_8 and Ling-NOR4_15. These metabolites can act against both fungal and bacterial pathogens [4,28]. *B. velezensis* Ling-NOR 4_15 carried the BGCs for plantazolicin, which is likely why it inhibited the growth of *F. culmorum* better than other bacteria. The gene *fenE*, involved in fengycin synthesis, was detected only in *B. halotolerans* Bil-LT1_2, which lethally affected the growth of *M. frutigena*. Notably, thailanstatin A, identified in both *B. halotolerans* strains (Bil-LT1_1 and Bil-LT1_2), has known antifungal and antitumor activities due to pre-mRNA splicing inhibition [29], while subtilosin A, a cyclic bacteriocin, is active against food borne gram-positive pathogens, like *S. aureus* and *L. monocytogenes* [30,31].

Beyond pathogen antagonism, functional annotation revealed a suite of plant growth-promoting (PGP) genes. These include genes linked to nutrient acquisition—phosphate transport (*pstB3*, *pstA*, *pstS*), nitrogen uptake and assimilation (*nasE*, *nasD*, *nifS*, *rocG*) and iron acquisition (*fhuD*, *fhuG*), as well as hormonal modulation due to tryptophan biosynthesis (*trpABCDEF*) linked to indole-3-acetic acid (IAA) production. Additionally, stress responce genes (*aldH*, *amiE*, *sufU*) and those involved in volatile organic compound (VOC) biosynthesis *(alsD*, *ilv* operon components) suggest potential for promoting plant vigor, stress tolerance, and induced systemic resistance [32,33,34,35,36,37]. The observed improvement in in *Fragaria* × *ananassa* cv. Elsanta yield and quality under bacterial inoculation further supports their agronomic relevance [38].

A particularly compelling insight from this study is the high number of horizontally acquired genes (HGTs) in these *Bacillus* genomes, especially in *B. halotolerans* strains. HGT is a key mechanism driving endophyte adaptability, plays a pivotal role in shaping the genome dynamics, enabling acquisition of novel traits under selective pressure from the host plant environment, microbial competitors, and abiotic stressors [16]. Genomic plasticity conferred by HGT enhances survival, niche occupation, and metabolic versatility traits crucial for long-term endophytic residency [39].

Comparative genomic analysis revealed mosaic genome structures enriched with mobile genetic elements and integrated islands associated with different Gene Ontology (GO) pathways relevant to nutrient metabolism, secondary metabolite biosynthesis, and stress response. HGT mechanism contributes to genome plasticity, equipping endophytes with genes for antibiotic resistance, virulence modulation, and secondary metabolite biosynthesis [20,40,41]. Previous studies have shown that endophytes in wild or long-lived perennials often harbour more HGT-acquired traits, facilitating co-evolution with their plant hosts [39,42]. GO enrichment of HGT regions highlighted processes such as nitrogen metabolism, biosynthetic processes, and aromatic compound biosynthesis. *Vaccinium* spp. plants are adapted to nutrient-poor environments, particularly acidic soils where nitrogen availability is limited, and essential for endophytes was the Cellular Nitrogen Compound Metabolic Process (GO:0034641). It encompasses a wide range of biochemical pathways involved in the synthesis, utilization, and degradation of nitrogen-containing molecules. This process is crucial in bacteria and plants, particularly in relationships of endophytes, where nutrient exchange and metabolic cooperation are highly active [22]. Other pathways Enrichment Cellular Aromatic Compound Biosynthetic Process (GO:0019438) and Macromolecule Metabolic Process (GO:0043170), become competitive tools in polymicrobial environments and allow mutation tolerance, stress resistance, and even genetic control in foreign environments.

Functional advantages in pathways acquired through HGTEs provide a competitive advantage in polymicrobial environments, allowing for more efficient colonization, persistence, and coevolution with the host. It is even more evident when the host plant is bilberry, lingonberry or cranberry, which occupy exceptionally specific ecological niches, and their dependence on finely tuned host-microbe interactions, suggests that endophytes in these plants have undergone co-adaptation [43].

Emerging studies on endophyte–pathogen interactions further suggest a role for endophytes in modulating plant defence and microbial competition through direct antagonism or priming of host responses. As cultivation conditions are optimized and secondary metabolite production pathways are increasingly understood, these strains hold promise for integration into biocontrol strategies, sustainable agriculture, and plant microbiome engineering [16,39].

## 4. Materials and Methods

The research was carried out at the Lithuanian Research Centre for Agriculture and Forestry, Institute of Horticulture (Babtai, Lithuania), and Kaunas University of Technology Food Institute (Kaunas, Lithuania).

### 4.1. Microorganisms

The endophytic *Bacillus* spp. strains (Bil-LT1_1, Bil-LT1_2, Cran-LT1_8, and Ling-NOR4_15) from *Vaccinium* plans was obtained during project NovelBaltic in 2021 and identified through 16S rRNA gene sequencing, with funding from the European Regional Development Fund through Interreg Baltic Sea Region Program [17]. The bacterial strains are preserved in the microorganism collection of the Food Institute at Kaunas University of Technology (Kaunas, Lithuania) and in liquid nitrogen at the Cryopreservation laboratory at Lithuanian Research Centre for Agriculture and Forestry (LAMMC).

Reference strains of foodborne bacteria were sourced from the microorganism collection of the KTU Food Institute. Preparation of endophytic bacteria strains for inhibition experiment: 48 h before inhibition test strains were pre-cultivated on LB agar (Luria-Bertani agar, Liofilchem, Roseto degli Abruzzi, Italy) at 26 °C for 48 h.

Plant pathogenic fungal isolates were obtained from the fungal pathogen collection of the LAMMC Laboratory of Plant Protection. Preparation of fungal isolate for inhibition experiment: before inhibition test isolate was maintained in Petri dishes on PDA (Potato dextrose agar, Liofilchem, Roseto degli Abruzzi, Italy) at 22 °C for 7 days in the dark.

### 4.2. Antagonism Evaluation In Vitro

Method dual culture assay for antifungal activity of bacterial isolates applied and the antagonistic effect of bacterial isolates on the radial growth of phytopathogenic fungi in vitro evaluated. A 5 mm diameter plug from an actively growing fungal culture) was placed in centre of a Potato Dextrose Agar (PDA) Petri plate. A bacterial isolate was streaked in a straight line on the opposite side of the same plate, also 1 cm from the edge, directly facing the fungal plug. A separate plate containing only the fungal plug (placed at the same position) was used as a growth control. All plates were incubated at 25 °C until the fungal colony in the control plate nearly reached the edge of the dish. Each treatment was performed in triplicate, and mean values ± standard deviations were reported. Photographs were taken of each plate after incubation for visual documentation. Inhibition zones and colony edges were measured using ImJoy v0.11.39 software for precise quantification of fungi growth area. Percent of fungal zone change was calculated using the following formula: Percent change = (New growth zone − Control growth zone)/Control growth zone × 100.

The agar well diffusion method was used to determine the antibacterial activities of selected endophytic *Bacillus* spp. strains in three replicates. The cultures of reference strains *Listeria monocytogenes* ATCC 13932, *Bacillus cereus* ATCC 11778, *Salmonella. enterica* subsp. *enterica* serovar Typhimurium ATCC 14028, *Staphylococcus aureus* subsp. *aureus* ATCC 25923, and *Escherichia coli* ATCC 25922 were pre-cultivated on PCA (Plate Count Agar, Liofilchem, Italy) slants at 37 °C or 30 °C overnight. The grown-up bacterial cultures were washed off from the agar with sterile physiological saline solution (NaCl 0.85% in distilled water). The cell suspension of each culture was adjusted according to McFarland standard No 0.5 (corresponding to 1.5 × 10^8^ CFU/mL). Then, one milliliter of the prepared suspension was added into 100 mL of melted and cooled to 45 °C LB agar. The prepared mixture of bacteria cell suspension and the medium was mixed thoroughly and poured into Petri dishes (90 mm), 12 mL each. After the medium had solidified, wells of 8-mm diameter were made in the plates, which were filled with 50 μL of the endophytic *Bacillus* spp. strain suspensions (concentration ~10^6^ CFU/mL). Antibacterial activity of endophytic *Bacillus* spp. strains wasevaluated after 48 h of growth at 26 °C. The diameter of inhibition zones was measured with calipers to an accuracy of 0.5 mm. As a control in the blank sample, distilled water was used.

### 4.3. Nutritional Preferences Test In Vitro

*Bacillus* strains were evaluated for their nutritional preferences using six different culture media: LB agar (Luria-Bertani, Liofilchem, Italy), PCA (Plate count agar, Liofilchem, Italy), TSA (Tryptic Soy Agar, Liofilchem, Italy), MYP (Mannitol egg Yolk Polymyxin agar, Liofilchem, Italy), NA (Nutrient agar, Liofilchem, Italy) and SBA (Sheep Blood Agar, Liofilchem, Italy). Each medium was prepared according to the manufacturer’s instructions and poured into sterile Petri dishes. A 16-h culture of each *Bacillus* strain (~10^6^ CFU/mL) was streaked onto the surface of the media using a sterile loop under aseptic conditions. Inoculated plates were incubated at 26 °C for 48 h. Following incubation, bacterial growth was visually evaluated, and colony morphology, size, pigmentation, and overall growth density.

### 4.4. Genomic DNA Extraction, Whole-Genome Sequencing, Annotation

Genomic DNA from *Bacillus* strains (Bil-LT1_1, Bil-LT1_2, Cran-LT1_8, and Ling-NOR4_15) with broad-spectrum antimicrobial activity was extracted using a commercial DNA extraction kit (Quick-DNA HMW MagBead Kit, Zymo Research Europe, Freiburg, Germany), following the manufacturer’s instructions. DNA quality and concentration were evaluated with Nanodrop (Implen, Munchen, Germany) and agarose gel electrophoresis systems.

High-quality genomic DNA was sequenced by Novogene (Cambrige, UK) using the NovaSeq X Plus Series (PE150) sequencing platform (1 G raw data per sample). Raw reads were quality-filtered and assembled using SPAdes (v4.2.0). Sequences were assembled, and contigs were obtained using Unicycler software (v0.5.1) [44]. BUSCO analysis was used to assess the completeness of the genome assembly [45]. Cleaned sequences were deposited to NCBI, accession numbers: SAMN36292479, SAMN36292480, SAMN36292481 SAMN36292482.

Bacteria genome annotation was carried out using Bakta (1.11.0) [46]. Antibiotics and Secondary Metabolites Analysis SHell (antiSMASH v8.0) was employed to identify biosynthetic gene clusters (BGCs) [47]. The comprehensive antibiotic resistance database (CARD) [48] and the virulence factor database (VFDB) [49] were used for putative genes identification. The human pathogenic capacity prediction was checked with PathogenFinder2-0.5.0 [50], predictions range from 0 (without pathogenic capacity) to 1 (with pathogenic capacity). KOALA (KEGG ortholog and links annotation) was used for functional characterization of the genome [51,52].

### 4.5. Comparative Genomic and Phylogenetic Analysis

Assembled genomes were mapped, compared with publicly available reference genomes in NCBI of *B. halotolerans*: CP029364, CP126100, CP110264, CP098738, CP138201, CP136430, CP041357) and *B. velezensis* (CP009679, CP119675, CP011937, CP026610, CP168028, CP049904, CP034203) and visualized using a bioinformatic tool Proksee (v1.0.0) [52]. Putative Horizontal Gene Transfer (HGT) events prediction was done with AlienHunter (v1.3.0) [53]. Venn diagram constructed through https://bioinformatics.psb.ugent.be/webtools/Venn/ (accessed on 20 April 2025) and for graphical gene-set enrichment in GO pathways ShinyGO (v0.82) was used [54].

## 5. Conclusions

The endophytic *B. halotolerans* strains Bil-LT1_1, Bil-LT1_2, along with *B. velezensis* strains Cran-LT1_8, and Ling-NOR4_15, characterized in this study, exhibit a comprehensive suite of plant-beneficial traits: robust antimicrobial activity, metabolic adaptability, plant growth promotion capabilities, and genome plasticity driven by horizontal gene transfer. Their abilities to inhibit a broad range of microorganisms and thrive in diverse nutritional environments highlights their potential as possible biocontrol agents and biofertilizers for sustainable agriculture. Identified GO pathways (GO:0034641, GO:1901576, GO:0009058, GO:1901564, GO:0044249) and genes acquired through horizontal gene transfer are closely related to the ecological conditions and survival requirements of their host plants. This is particularly significant for *Vaccinium* spp. such as bilberry, cranberry or lingonberry, which are adapted to extremely poor and specific ecological niches. The observed genomic plasticity and functional versatility of these strains suggest a well-established endophytic lifestyle, in the internal leaf tissues of *Vaccinium* spp., offering systemic benefits to their host plants.

While the in vitro antagonistic activity of the characterized strains is promising, the absence of in planta validation remains a limitation of this study. Without testing under realistic plant-microbe and soil conditions, the practical application potential of these strains cannot be fully assessed. The findings suggest that these endophytic strains possess genes associated with antimicrobial activity and stress adaptation, which may be relevant for future applications in biocontrol, biofertilization, or environmental remediation, pending further experimental validation in plant or soil systems. Future studies should include in planta trials to evaluate colonization dynamics, bio-control efficacy, soil improvement capacity, and interactions with the native microbiome under greenhouse and field conditions.

## Figures and Tables

**Table 1:** Summary of antimicrobial activity and growth characteristics of *Bacillus* endophytes isolated from *Vaccinium* spp.

Strain	Bil-LT1_1	Bil-LT1_2	Cran-LT1_8	Ling-NOR4_15
**Antifungal Activity, Growth Suppression (%)**				
*B. cinerea* T11B_BRA_189	52.4 ± 1.7	69.1 ± 1.5	79.7 ± 1.1	48.5 ± 0.6
*F. culmorum* LT_1V23b	23.3 ± 0.7	19.2 ± 0.7	26.9 ± 0.5	29.7 ± 0.9
*M. fructicola* CBS 101512	50.6 ± 1.5	56.2 ± 0.3	59.4 ± 1.7	56.4 ± 0.8
*M. frutigena* MFS01	68.7 ± 1.8	99.1 ± 0.0	91.7 ± 0.3	77.5 ± 0.6
*M. laxa* CBS 489.50	61.2 ± 1.0	59.7 ± 1.1	84.8 ± 0.5	68.8 ± 1.5
**Antibacterial Activity** **, Inhibition Zone (mm)**				
*B. cereus* ATCC 11778	10.0 ± 0.1	10.0 ± 0.1	16.0 ± 0.1	16.0 ± 0.1
*E. coli* ATCC 25922	N/A	N/A	12.0 ± 0.1	N/A
*L. monocytogenes* ATCC 13932	11.0 ± 0.2	12.0 ± 0.2	16.0 ± 0.1	16.0 ± 0.1
*S. enterica* subsp. *enterica serovar Typhimurium* ATCC 13932	N/A	N/A	14.0 ± 0.1	11.0 ± 0.0
*S. aureus* subsp. *Aureus* ATCC 25923	14.0 ± 0.3	11.0 ± 0.1	18.0 ± 0.3	14.0 ± 0.1
Growth on media *	Good on all tested media (LB, PCA, TSA, NA, MYP)	Dense colonies on PCA; good on other media	Large, slightly mucoid colonies on PCA; low density on TSA; poor on NA	Uniform growth on NA; pink hue on MYP
**Hemolysis on SBA**	Beta	Beta	Alpha/Beta	Beta
**Mannitol fermentation on MYP**	Positive (orange/yellow shift)	Positive (orange/yellow shift)	Weak/partial (yellowish; inhibited growth)	Negative (pink hue)
**Pigment production**	Yellow on TSA	None observed	None observed	None observed

* LB—Luria-Bertani agar; PCA—Plate Count Agar; TSA—Tryptic Soy Agar; NA—Nutrient agar; MYP—Mannitol egg Yolk Polymyxin agar; SBA—Sheep Blood agar.

**Table 2:** The main statistics of sequencing, mapping data and assembly—annotation information of endophytic *Bacillus* strains from *Vaccinium* spp. plants.

	Isolated *B. halotolerans*		Isolated *B. velezensis*	
	Bil-LT1_1	Bil-LT1_2	Cran-LT1_8	Ling-NOR4_15
	SAMN36292479	SAMN36292480	SAMN36292481	SAMN36292482
**Statistics of Sequencing Data**				
Raw reads	4,383,886	4,967,478	4,499,288	4,914,838
Raw data (G)	1.1	1.2	1.1	1.2
Clean data (G)	1	1	1	1.1
Effective (%)/Error (%)	91.01/0.04	85.39/0.03	85.18/0.04	86.38/0.04
GC (%)	43.3	43.3	46.3	46.3
**Assembly Information**				
Total reads	4,259,074	4,261,465	4,170,815	4,593,628
Number of contigs	39	34	28	21
Largest contig (bp)	646,884	646,885	940,386	941,931
**Mapping Statistics to the Reference Genomes**				
	CP029364 (NCBI)		CP119675 (NCBI)	
Average nucleotide identity (ANI), %	99.50	99.82	98.01	97.95
**Annotation Information**				
Bakta annotation	4472	4472	3962	4025
CDSs	4313	4313	3805	3897
tRNAs; tmRNAs; rRNAs	62; 1; 4	62; 1; 4	73; 1; 3	77; 1; 3
ncRNAs	28	28	19	21
ncRNA regions	62	62	59	60
Proteins with KEGG pathway assignments	2457	2458	2315	2317
**Specialty Genes**				
Antibiotic resistance (CARD)	13	13	7	7
Virulence factors (VFBDs)	39	39	38	38
Biosynthetic gene clusters (BGCs)	14	14	20	21
Biosynthesis of antibiotics	5	5	3	2
**Bacterial Pathogenic Capacity Prediction**				
Mean (std)	0.0942 (0.0204)	0.0825 (0.0181)	0.0514 (0.0163)	0.0511 (0.0191)

**Table 3:** Share and unique secondary metabolites in *B. halotolerans* and *B. velezensis* strains.

Bacteria Strain	Clusters for Secondary Metabolites Synthesis
Bil-LT1_1, Bil-LT1_2 Cran-LT1_8, Ling-NOR 4_15	plipastatin, bacillaene, teichuronic acid, chejuenolide A/chejuenolide B, K53 capsular polysaccharide, bacillomycin D, surfactin, dipeptide aldehydes, fengycin, bacillibactin, bacilysin
Bil-LT1_1, Bil-LT1_2	thailanstatin A, subtilosin A
Cran-LT1_8, Ling-NOR 4_15	butirosin A/butirosin B, molybdenum cofactor, difficidin, macrolactin H
Ling-NOR 4_15	plantazolicin
Cran-LT1_8	mersacidin, rhizomide A/rhizomide B/rhizomide C

**Table 4:** Plant growth promotion genetic features.

Function	*B. halotolerans*		*B. velezensis*	
	Bil-LT1_1	Bil-LT1_2	Cran-LT1_8	Ling-NOR 4_15
	Genes			
**Phosphate metabolism**	*pstB3*, *pstA*			
	*pstS*	*-*	*pstS*	*pstS*
**Nitrogen metabolism**	*nasE*, *nasD*, *rocG*			
**Nitrogen fixation**	*nifS*, *salA*, *sufU*			
**Siderophore**	*fhuD*, *pchA*			
	*-*	*fhuG*	*-*	*-*
**Phytohormone production**	*trpA*, *trpB*, *trpF*, *trpC*, *trpD*, *trpE*, *amiE*, *aldH*			
**Hydrolase**	*amyE*, *eglS*, *gmuD*, *ganB*			
**Chitinase activity**	*sleL*, *ydhD*			
**Biofilm**	*tasA*, *bslA*			
	*bslB*	*bslB*	*-*	*bslB*
**2,3-butanediol**	*alsD*, *ilvK*, *ilvE*, *ilvA*, *ilvD*, *ilvC*, *ilvH*, *ilvB*			

**Table 5:** Comparative analysis of Horizontal Gene Transfer Elements (HGTEs) in different strains of *B. halotolerans* and *B. velezensis*.

Access. No. in NCBI and Strain Name		HGTEs Size Range bp	No of HGTEs	Information About Isolate	Genome Size, bp
***Bacillus halotolerans***					
SAMN36292479	Bil-LT1_1	5000–37,501	123	Bilberry leaves, Lithuania	4,259,074
SAMN36292480	Bil-LT1_2	5000–52,501	107	Bilberry leaves, Lithuania	4,261,465
CP029364	ZB201702	5131–63,099	61	Drought and salt rhizosphere soil of maize, China: Inner Mongolia	4,154,245
CP126100	LN2	4055–128,846	58	Rhizosphere soil, China: Longnan, Gansu Province	4,252,134
CP110264	HMB20199	4640–47,395	54	Plant, against *Pseudoperonospora cubensis* Rostow, China: Baoding	4,175,808
CP098738	XE48	6651–66,933	62	Marine low temperature surface sediment, West Indian Ocean	4,140,100
CP138201	DY299	5023–74,450	46	Soil, China: Inner Mongolia	4,208,811
CP136430	Q2H2	5970–56,767	49	Endophytic bacteria of potato root with strong antagonisticactivity, China: Inner Mongolia	4,155,130
CP041357	F41-3	5001–50,720	55	Flower of Chiness redbud, South Korea: Jeon-buk	4,144,458
***Bacillus velezensis***					
SAMN36292481	Cran-LT1_8	5001–35,001	50	Cranberry leaves, Lithuania	3,958,789
SAMN36292482	Ling-NOR 4_15	5001–35,001	56	Lingonberry leaves, Norway	3,987,686
CP009679	JS25R	3864–33,478	41	Spikelet of wheat heads, with biocontrol activity China: Beijing	4,006,002
CP119675	160	5156–135,917	28	Rhizosphere soil of corn crops, control agent of corn head smut, Mexico	4,296,610
CP011937	CBMB205	4737–36,609	48	Rice rhizosphere soil, South Korea: Cheongwon	3,929,792
CP026610	CGMCC 11640	5090–142,965	50	Bamboo forest soil, against Botryosphaeria dothidea, China:Tianmu Mountain	4,322,979
CP168028	CM5	3808–140,634	56	Peat casing layer, biocontrol in the mushroom microcosm Spain: Rioja	4,240,819
CP049904	GB03	4777–34,819	33	Biological Control Product Kodiak TM, Korea	3,894,540
CP034203	83	3879–36,447	35	Biological Control Product Fungifree AB™, Korea	3,997,902

**Table 6:** Venn diagram and HGTEs annotation in *Vaccinium* spp. endophytic strains.

** 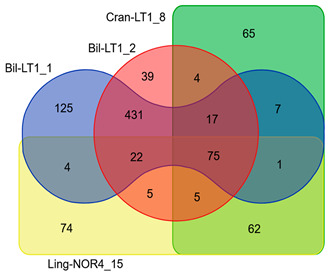 **	**List Strains**	**Total Number of CDS**	**Number of Annotated Genes**	**Antibiotic Resistance Genes**	**Virulence Factors**
	Bil-LT1_1	1155	682	*satA_Bs*, *rphC*	14 *
	Bil-LT1_2	1095	598	*satA_Bs*, *rphC*	13 **
	Cran-LT1_8	525	236	*rphC*, *tet(L)*, *satA_Bs*	*cheY*
	Ling-NOR4_15	605	248	*satA_Bs*, *tet(L)*, *clbA*, *rphC*	*dhbF*, *mbtH*

* Polysaccharide capsule coding gene, *dhbA,B,C,E,F*, *csrA*, *cdsN*, *ureB*, *wcaJ*, Capsule (Enterococcus) coding gene, *cbrD*, *mbtH*, *lgt*. ** Polysaccharide capsule coding gene, *dhbA,B,C,E,F*, *csrA*, *cdsN*, *wcaJ*, Capsule (Enterococcus) coding gene, *cbrD*, *mbtH*, *lgt*.

## Data Availability

Data is contained within the article, Supplementary Materials and in NCBI database.

## Supplementary Materials

The following supporting information can be downloaded at: https://www.mdpi.com/article/10.3390/ijms26146677/s1.
