# Supplementary figures and images for: Genomic Insights into Vaccinium spp. Endophytes B. halotolerans and B. velezensis and Their Antimicrobial Potential
Source: Int J Mol Sci. 2025 Jul 11;26(14):6677. doi: 10.3390/ijms26146677 (PMC12294466; doi:10.3390/ijms26146677)

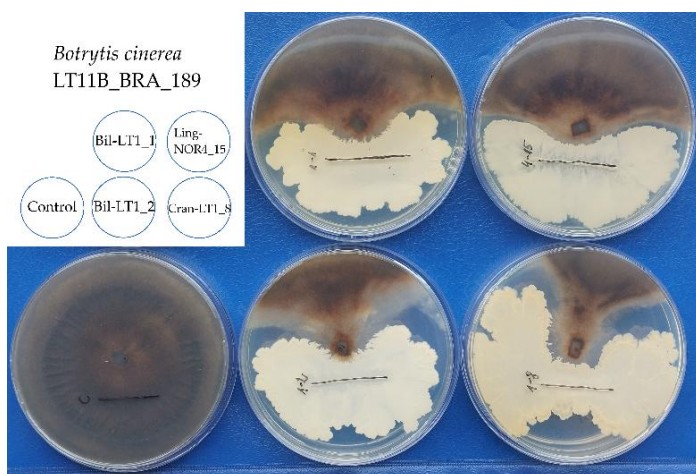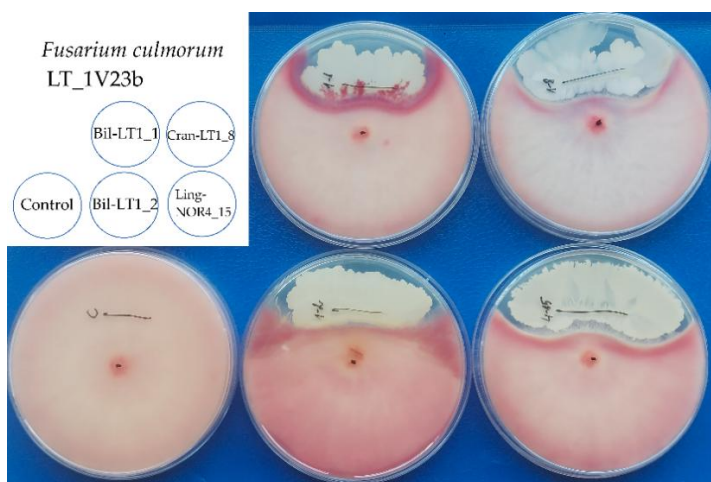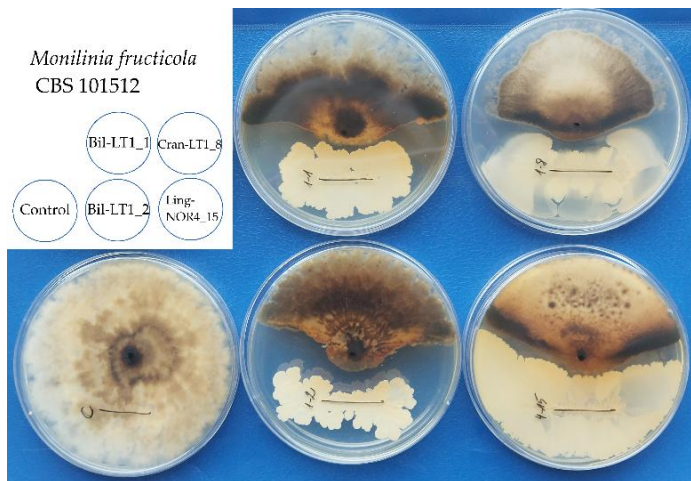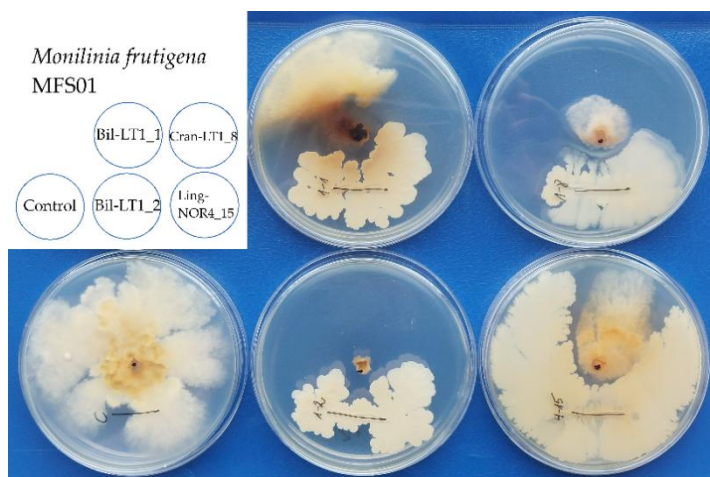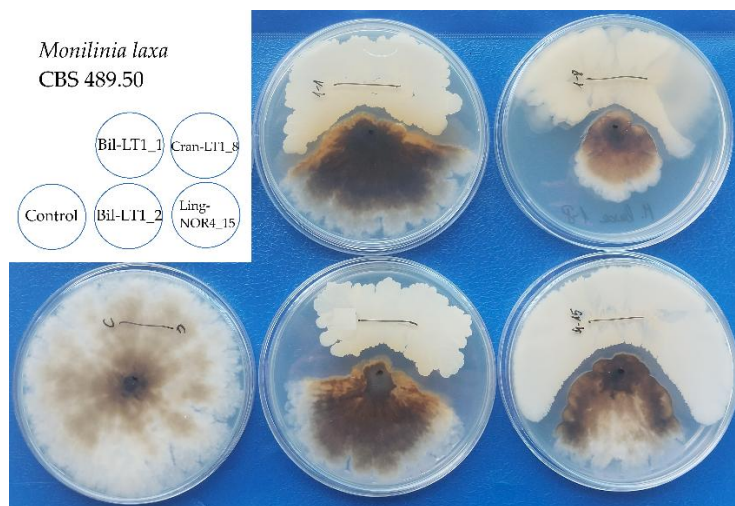

Figure S1. Fungi growth suppression on potato dextrose agar (PDA) plate (90 mm).

Supplement: Supplementary file 1 [file ijms-26-06677-s001.zip › ijms-3708904_Supplements/Figure_S1.pdf]

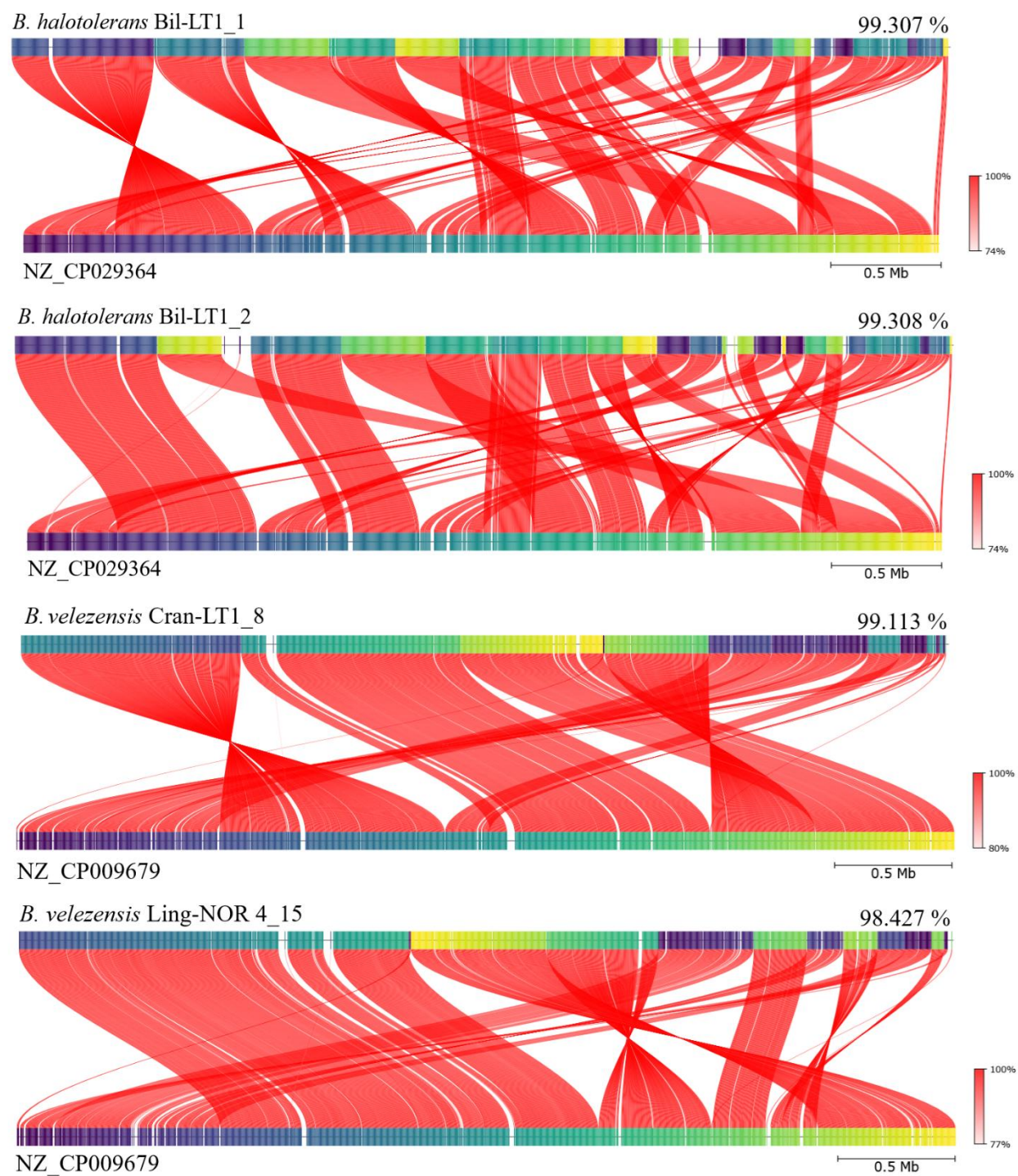

Fig. S2. Average nucleotide identity (ANI) with reference genomes.

Supplement: Supplementary file 1 [file ijms-26-06677-s001.zip › ijms-3708904_Supplements/Figure_S2.pdf]
